# Burden of Invasive Pneumococcal Disease in Children Aged 1 Month to 12 Years Living in South Asia: A Systematic Review

**DOI:** 10.1371/journal.pone.0096282

**Published:** 2014-05-05

**Authors:** Nishant Jaiswal, Meenu Singh, Kiran Kumar Thumburu, Bhavneet Bharti, Amit Agarwal, Ajay Kumar, Harpreet Kaur, Neelima Chadha

**Affiliations:** 1 ICMR Advanced Centre for Evidence Based Child Health, Post Graduate Institute of Medical Education and Research, Chandigarh, India; 2 Department of Pediatrics, Post Graduate Institute of Medical Education and Research, Chandigarh, India; 3 Tulsidas Library, Post Graduate Institute of Medical Education and Research, Chandigarh, India; 4 University Business School, Punjab University, Chandigarh, India; 5 Department of Ophthalmology, Wayne State University, Detroit, Michigan, United States of America; Aga Khan University, Pakistan

## Abstract

**Objective:**

The primary objective was to estimate the burden of invasive pneumococcal disease (IPD) in children aged 1 month to 12 years in South Asian countries.

**Methods:**

We searched three electronic databases (PubMed, Embase and the Cochrane Library) using a comprehensive search strategy, we manually searched published databases (Index Medicus and Current Contents) and we also searched the bibliographies of the included studies and retrieved reviews. The searches were current through June 2013. Eligible studies (community-based and hospital-based) were pooled and a separate analysis for India was also completed. A meta-regression analysis and heterogeneity analysis were performed. The protocol was registered with PROSPERO registration number CRD42013004483.

**Results:**

A total of 22 studies surveying 36,714 children were included in the systematic review. Hospital-based prospective studies from South Asia showed that 3.57% of children had IPD, and 15% of all bacterial pneumonia cases were due to *Streptococcus pneumoniae*. Indian studies showed that the incidence of IPD was 10.58% in children admitted to hospitals with suspected invasive bacterial diseases, and 24% of all bacterial pneumonia cases were due to *S. pneumonia*. Population-based studies from South Asian countries showed that 12.8% of confirmed invasive bacterial diseases were caused by *S. pneumonia* whereas retrospective hospital-based studies showed that 28% of invasive bacterial diseases were due to *S. pneumoniae*. Meta-regression showed that there was a significant influence of the antigen testing method for diagnosing IPD on IPD prevalence.

**Conclusion:**

*S. pneumoniae* is responsible for a substantial bacterial disease burden in children of South Asian countries including India despite the presence of high heterogeneity in this meta-analysis. Treatment guidelines must be formulated, and preventive measures like vaccines must also be considered.

## Introduction

Worldwide, pneumonia is the leading infectious cause of mortality in children under five years of age [1]. Besides pneumonia, meningitis and sepsis are also amongst the top killers of children in the world [1]. World Health Organization (WHO) estimates have shown that more than 90% of all deaths due to pneumonia in children under the age of five occur in 40 countries. The most deaths caused by pneumonia occur in India, with Pakistan, Bangladesh and Afghanistan following close behind [2]. Morris et al. predicted that the mortality rates of children under five years of age attributable to pneumonia are similar in Sub-Saharan Africa and South Asia [3].

According to estimates, more than half of the new cases of clinical pneumonia are concentrated in five countries, three of which are in South Asia, i.e., India, Bangladesh and Pakistan [4]. This core of South Asian countries has a high incidence of clinical pneumonia, with India having the highest number of new cases detected worldwide. The mortality rates for children under five years of age in the South Asian region ranges from 17/1000 for Sri Lanka to 149/1000 for Afghanistan. Pneumonia claims the lives of 11% of children under the age of five in India, the Maldives, Bangladesh and Pakistan, 23% in Afghanistan, 19% in Bhutan and 6% in Sri Lanka [5].

Modern evidence on the aetiology of pneumonia is based on two types of studies. The first type is hospital-based prospective studies, which rely on culture methods, and the second type is vaccine trials, which measure the reduction in the burden of a disease by specific vaccines against the disease [6], [7]. Both types of studies have found *Streptococcus pneumoniae* to be the leading cause of pneumonia. *S. pneumoniae* is an encapsulated diplococcus with over 90 known capsular serotypes. It causes a wide spectrum of diseases ranging from fatal invasive diseases like pneumonia, meningitis and sepsis to noninvasive diseases like otitis media and sinusitis [8]. *S. pneumoniae* affects both extremes of age, i.e., children and the elderly. South Asian countries have the highest incidence of mortality due to invasive pneumococcal disease (IPD) [8]. Moreover, *S. pneumoniae* infections are the leading vaccine preventable causes of mortality in children under the age of five worldwide [8].

Although vaccines against *S. pneumoniae* are available in South Asian countries, the majority have not included them in their respective immunization schedules. This omission is due to the paucity of data available on the burden of pneumococcal diseases and the lack of information on the prevalent *S. pneumoniae* serotypes in this region. Hence, this systematic review was conducted to provide these data.

## Objectives

The primary objective of this systematic review is to determine the pneumococcal disease burden in children aged 1 month to 12 years in South Asian countries. These data can help policymakers make decisions regarding the need for including pneumococcal vaccines in national immunization schedules.

## Methods

### Inclusion Criteria

1. 1 Published Studies: prospective or retrospective studies that included children aged 1 month to 12 years from South Asian countries.
2. 2 Isolation of *S. pneumoniae* from included subjects.
3. 3 Minimum 12 months of surveillance.

Two authors decided upon the inclusion of studies, and others performed quality assessment. Discrepancies, if any, were resolved by discussion.

If the required data were not available, we contacted the authors and attempted to retrieve the missing data. Studies that commented only on pneumococcal serotypes and/or antibiotic resistance were excluded from the pooled analysis. We excluded case reports, editorials, vaccine studies, literature reviews and studies in which nasopharyngeal aspirates, throat swabs or oropharyngeal swabs were the only samples used to determine the causative organism.

### Literature Search

We performed a systematic search of the published literature and also tried to acquire unpublished data from various investigators of the region. The searches were current as of June 2013. We identified articles with information on IPD amongst children aged 1 month to 12 years. We searched three databases: Medline via Ovid and PubMed, Embase and The Cochrane Library. The reference lists of the obtained articles were further searched for additional studies. Published databases (Index Medicus and Current Contents) were also manually searched. Non-English articles were not included. The search details are given in Appendix S1. Searching was performed independently by two authors.

### General Definitions

**South Asia** includes Afghanistan, Pakistan, India, Nepal, Bhutan, Bangladesh, Sri Lanka and the Maldives.

**Burden of pneumococcal disease**: We defined the burden of pneumococcal disease as the number of positive pneumococcal isolates from the suspected population (children aged 1 month to 12 years).

**Hospital Based Surveillance Studies** are those, which involve monitoring a disease at a single facility or small number of facilities.

**Population Based Surveillance Studies** are those, which involve identifying all new cases of the disease under surveillance in a defined population.

### Disease Definitions

1. 1 Pneumonia [9]

Symptoms: cough or difficult breathing. Signs: respiratory rate >50 breaths per minute for infants aged two months to less than one year, respiratory rate >40 breaths per minute for children aged one to five years, and no chest indrawing, stridor or danger signs.

1. 2 Severe pneumonia [9]

Symptoms: cough or difficult breathing plus any general danger sign, chest indrawing or stridor in a calm child. General danger signs for children aged two months to five years include the inability to drink or breastfeed, vomiting, convulsions and lethargy or loss of consciousness.

1. 3 Meningitis [9]
  - **Suspected**: Any person with sudden onset of fever and one of the following signs: neck stiffness, altered consciousness or other meningeal sign.
  - **Probable**: A suspected case with cerebrospinal fluid (CSF) examination showing at least one of the following:
    - turbid appearance;
    - leukocytosis (>100 cells/mm^3^);
    - leukocytosis (10–100 cells/mm^3^) and either elevated protein (>100 mg/dl) or decreased glucose (<40 mg/dl).
  - **Confirmed**: A case that is laboratory-confirmed by growing (i.e., culturing) or identifying (i.e., using antigen detection methods) a bacterial pathogen (pneumococcus) in the CSF or blood of a child with a clinical syndrome consistent with bacterial meningitis.
2. 4 Nonpneumonia nonmeningitis

All infections other than pneumonia and meningitis were categorized under this heading.

1. 5 Invasive pneumococcal disease

When pneumococcus is identified from one of the otherwise sterile sites of the body, such as blood, CSF or pleural fluid, either by culture, the latex agglutination test (LAT), polymerase chain reaction (PCR) or another technique.

### Data Collection and Management

Four authors independently abstracted data from the included studies in a predesigned *pro forma* that included study design, setting, number of suspected cases, site for sample collection, number of samples collected for culture, number of cultures positive for pneumococcus and prior use of antibiotic. The data from hospital-based and population-based studies were abstracted separately. Authors were contacted to obtain missing data. To resolve discrepancies regarding the abstracted data, a consensus was drawn by discussion with the other reviewers. If discrepancies were not resolved, the data were not included in the pooled analysis.

### Data Analysis

Data analysis was performed using STATA-MP 12, two-core, which is manufactured by Stata Corp., Ltd. Similar data from the studies were pooled for analysis. We calculated the proportion of pneumococcus isolated from the total bacterial isolates of the study population. The proportion of invasive pneumococcal disease from total suspected population was calculated using the total number of children with suspected invasive disease in the study as the denominator and for calculating the proportion of invasive pneumococcal disease from invasive bacterial disease the denominator used was the number of children with positive bacterial isolates either by culture or antigen testing. Additional analysis was performed to calculate the incidence of IPD in South Asian children less than 5 years of age and in Indian children aged 1 month to 12 years. Subgroup analysis was performed for pneumonia and meningitis. Data from hospital-based prospective studies, hospital-based retrospective studies and community-based studies were analysed separately. We calculated I^2^, Tau^2^ and the Z test for heterogeneity amongst the studies, and the Galbraith plot was used to represent the heterogeneity. To analyse publication bias, Begg's test, Kendall's score, Egger's linear regression and the funnel plot were used.

## Results

### Data Reviewed

**Figure 1:**
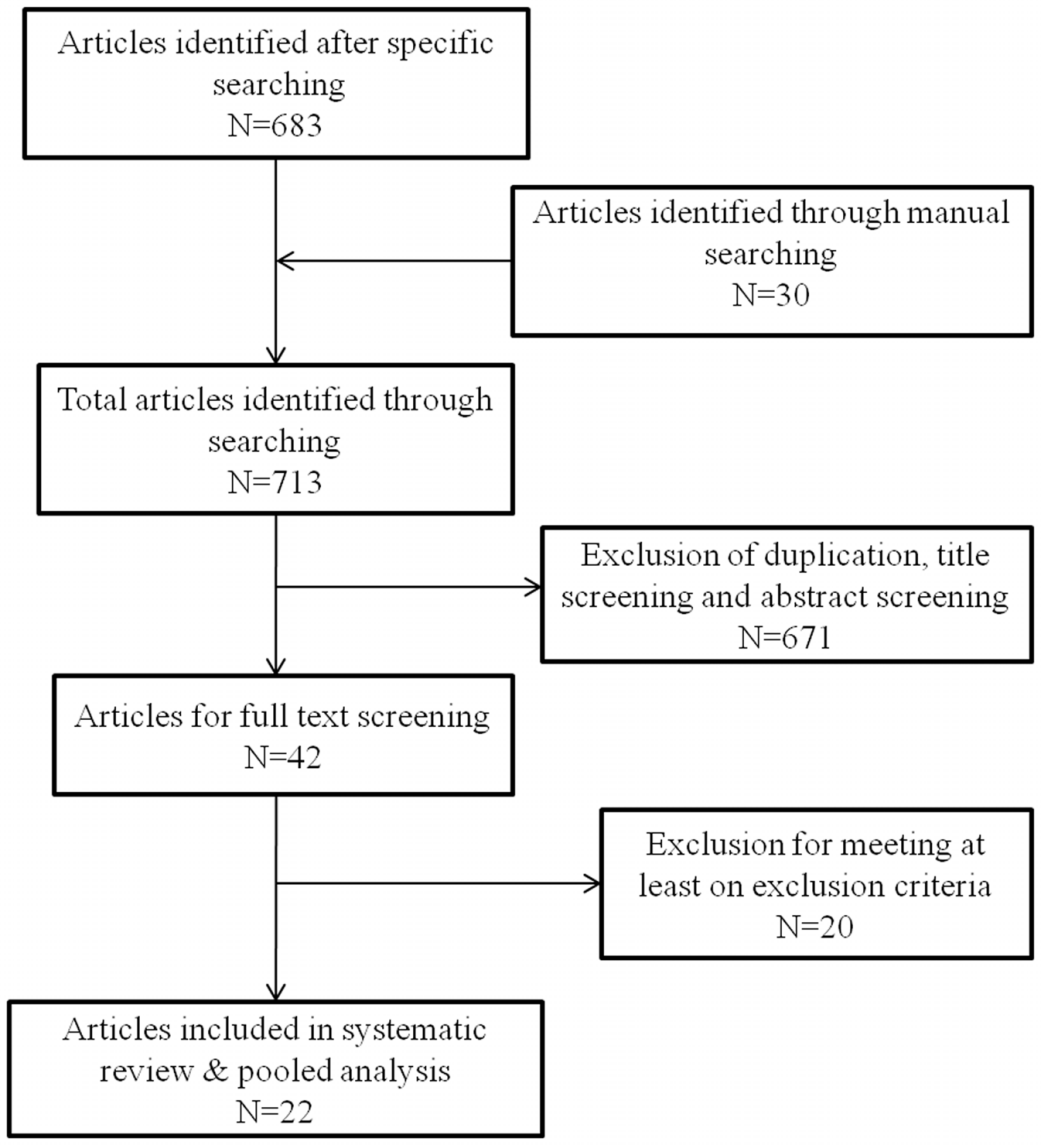
Flow diagram of search results.

We found 713 published articles through electronic and manual searches. After screening the titles and abstracts, 42 full-text articles were retrieved, of which 22 studies fulfilled the inclusion criteria and were included in the analysis (Figure 1, Table 1) [10]–[31]. Twenty studies were excluded from the systematic review [32]–[51], as shown in Table 2. The total number of children surveyed was 36,714. Of these subjects, 2,539 children were confirmed to have a bacterial disease. A total of 663 children less than 12 years of age were confirmed to have IPD. A detailed analysis of each study type was completed separately and is described below.

**Table 1:** Characteristics of included studies.

Serial no.	Country (ref)	Setting	Study year	Duration (months)	Syndromes/diseases studied	Diagnostic method	Prior antibiotic usage	Population (age)	Number of positive bacterial growth	Number of cases with *S. pneumoniae*
1	India [10]	Hospital based prospective study	Sep'78–Feb'81	29	Meningitis	CSF gram stain, culture & CIEP	Reported prior antibiotic usage	70 (<15 years)	29	13
2	India [11]	Hospital based prospective study	Feb'85–Dec'87	23	Pneumonia	Blood culture	Did not report prior antibiotic usage	331 (<6 years)	28	8
3	India [12]	Hospital based prospective study	Jan'89–Apr'90	16	Meningitis	CSF culture, Gram stain and LAT	Did not report prior antibiotic usage	114 (2 mth-11 yr)	55	15
4	India [13]	Hospital based prospective study	Sep'88–Aug'89	12	Pneumonia	Blood culture, Throat swab and NPA culture	Did not report prior antibiotic usage	132 (<12 years)	34	13
5	India [14]	Hospital based prospective study	1993–1997	48	Pneumonia, Meningitis, Septicemia	Blood culture and CSF culture	Reported prior antibiotic usage	5738 (<12 yrs)	N.A.	156
6	India [15]	Hospital based prospective study	Sep'94–Apr'96	20	Meningitis	CSF culture, cel count, biochemistry and LAT	Reported prior antibiotic use	100 (1mth – 12 yrs)	35	12
7	India [16]	Hospital based prospective study	Mar'95–Feb'97	24	Pneumonia	Blood culture, Throat swab and NPA culture	Did not report prior antibiotic usage	95 (2mths - 5 yrs)	15	5
8	India [17]	Hospital based prospective study	Jul'00–Jul'01	12	Meningitis	CSF culture	Reported prior antibiotic use	150 (<12 yrs.)	40	6
9	Bangladesh [18]	Population based prospective study	1999–2001	24	Pneumonia	Blood culture	Did not report prior antibiotic usage	18,983 (<5 years)	331(840)	7
10	Bangladesh [19]	Population based prospective study	2004–2006	24	Severe Pneumonia	Blood culture	Did not report prior antibiotic usage	6167 (<5 years)	315(5949)	34
11	India [20]	Hospital Based retrospective study	Jan'96–Dec'05	120	Meningitis	Records, CSF culture & LAT	Did not report prior antibiotic usage	51 (<12 years)	40	28
12	India [21]	Hospital based prospective study	Feb'03–Jan'07	48	Meningitis	CSF culture & LAT	Reported prior antibiotic use	535 (<5 years)	214	94
13	Bangladesh [22]	Population based prospective study	Jul'04–Jun'07	36	Pneumonia, Meningitis, Septicemia	Blood & CSF culture	Did not report prior antibiotic usage	6966 (<5 years)	93	26
14	Srilanka [23]	Hospital based prospective study	Jan'05–Mar'07	27	Pneumonia, Meningitis, Septicemia	Blood & CSF culture	Reported prior antibiotic use	3642 (<5 years)	585	37
15	Bangladesh [24]	Hospital based prospective study	May'04–Apr'07	36	Pneumonia	Blood culture	Reported prior antibiotic use	4155 (<5 years)	161	10
16	Nepal [25]	Hospital based prospective study	Apr'05–Dec'06	21	Pneumonia, Meningitis, Septicemia	Blood & CSF cultures	Reported prior antibiotic use	885 (<5 years)	47	17
17	Nepal [26]	Hospital based prospective study	Nov'04–Mar'07	29	Pneumonia, Meningitis, Septicemia	Blood & CSF cultures	Reported prior antibiotic use	2529 (<5 years)	276	51
18	India [27]	Hospital Based retrospective study	Jan'06–Dec'06	12	Pneumonia, Meningitis, Septicemia	Records	Did not report prior antibiotic usage	2219 (<5 years)	N.A.	61
19	Pakistan [28]	Hospital based prospective study	May'05–Apr'06	12	Meningitis	CSF culture	Reported prior antibiotic use	2690 (<5 years)	83	32
20	Pakistan [29]	Population based prospective study	2007–2008	15	Pneumonia	Blood cultures	Did not report prior antibiotic usage	5570 (<5 years)	36(1147)	1
21	Nepal [30]	Hospital based prospective study	Apr'05–Dec'06	21	Pneumonia, Meningitis, Septicemia	Blood and CSF culture	Reported prior antibiotic use	2039 (<12 years)	151	36
22	India [31]	Hospital based prospective study	Sep'91–Jul'92	12	Pneumonia	Blood culture, LAT	Reported prior antibiotic use	110 (<5 years)	62	32

**Table 2:** Characteristics of excluded studies.

S. no	Study name	Comments
1.	Patwari et al, 1988 [46]	No available data on causative organism
2.	Mastro et al 1991 [41]	Antibiotic resistance only
3.	Mastro et al,1993 [42]	Study period is <1 year, Nasopharyngeal aspirates only
4.	Awasthi et al 1997 [35]	No data on *S. pneumoniae*
5.	Saha et al 1997 [48]	Serotype details only
6.	Saha et al 1999 [49]	Mentions about antibiotic resistance only
7.	Jebaraj et al 1999 [40]	Nasopharyngeal colonization study
8.	*Addo-Yobo et al* 2004 [50]	Randomized controlled trial, has mixed data of Asia and Africa and South America also
9.	Acharya et al 2003 [32]	Does not report for *S. pneumoniae*
10.	Mehta et al,2003 [44]	Tells about Antibiotic resistance only does not give the details of *S. pneumoniae* and other causative organism
11.	Bansal et al, 2006 [36]	Not reported *S. pneumoniae* so cannot be included
12.	Bharti et al, 2006 [38]	No information on *S. pneumoniae*
13.	Hussain et al 2006 [39]	Cost of treatment study
14.	Nizami et al 2006 [45]	Oropharyngeal aspirate only
15.	SPEAR study 2008 [34]	Study does tell only about India but has included other regions which are not a part of South Asia, Randomized Control Trial
16.	Agarwal et al, 2009 [33]	Short report; No data for *S. pneumoniae*
17.	Saha et al 2009 [51]	Serotype details only
18.	Rijal et al 2010 [47]	Serotype details only
19.	Mathisen et al 2010 [43]	Study on viruses
20.	Bansal et al, 2004 [37]	Study of adults

### Hospital-based Prospective Studies

#### South Asian Countries: (children aged 1 month to 12 years)

**Figure 2:**
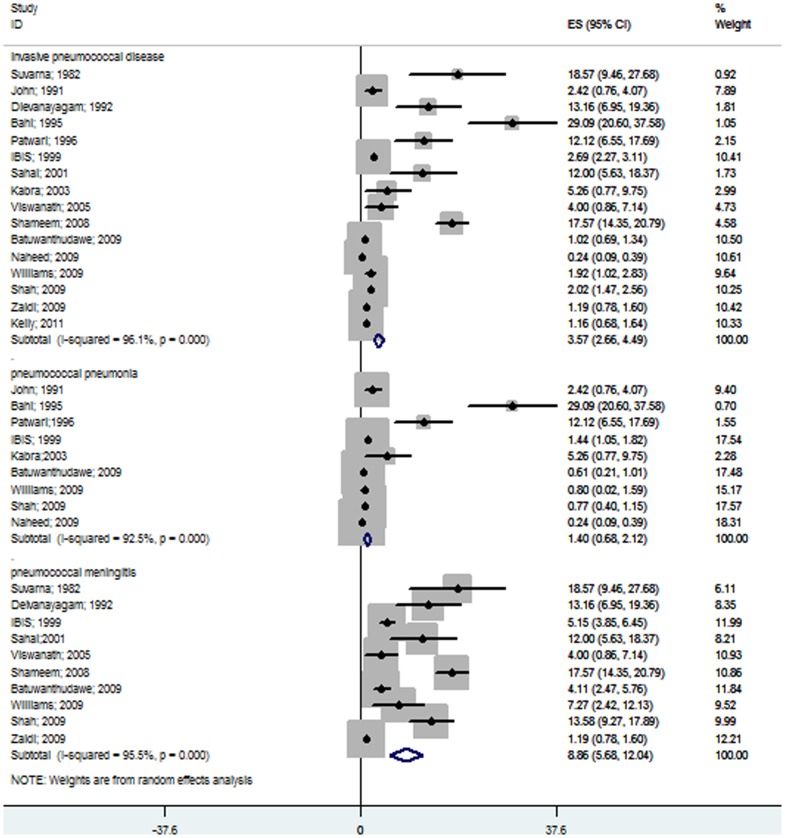
Forest plot showing the proportion of IPD from hospital-based prospective studies on South Asian children aged 1 month to 12 years with suspected invasive bacterial disease. The plot also shows the subgroup analysis for the pneumococcal pneumonia cases among all of the pneumonia patients and for the pneumococcal meningitis cases among all of the meningitis patients.

**Figure 3:**
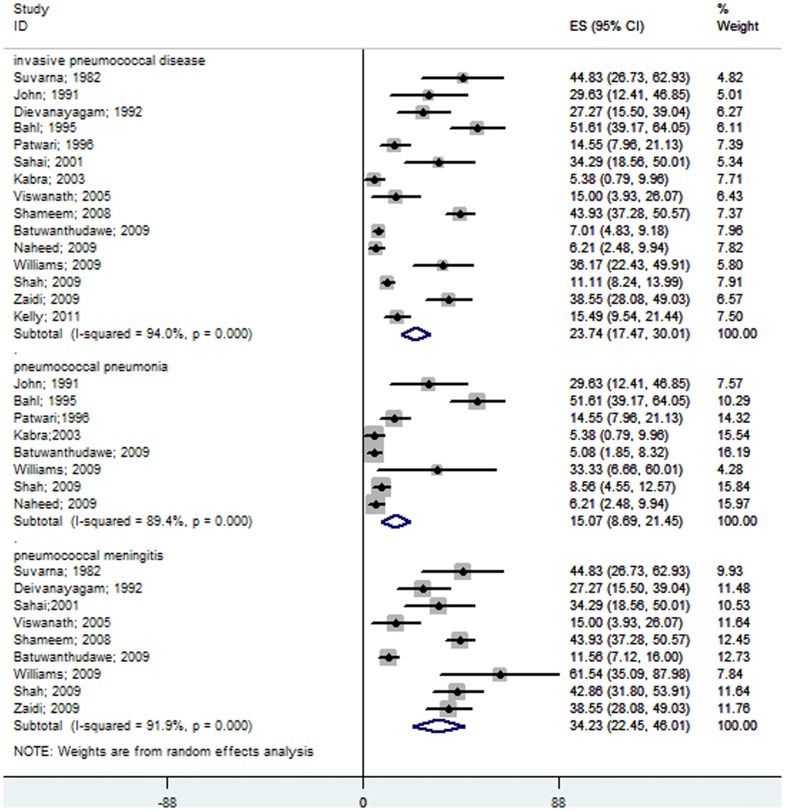
Forest plot showing the proportion of IPD from hospital-based prospective studies in South Asian children aged 1 month to 12 years with confirmed invasive bacterial disease. The plot also shows a subgroup analysis for the pneumococcal pneumonia cases among all of the bacterial pneumonia patients and for the pneumococcal meningitis cases among all of the pyogenic meningitis patients.

We identified sixteen hospital-based prospective studies from various South Asian countries [10]–[17], [21], [23]–[26], [28], [30], [31], and the pooled analysis using the random effects model showed that 3.57% (95% CI 2.66–4.49) of children admitted to hospitals with suspected invasive bacterial diseases were due to *S. pneumoniae*. Nine studies showed that only 1.4% (95% CI 0.68–2.12) of children admitted with severe pneumonia were due to pneumococcus (Figure 2) [11], [13], [14], [16], [23]–[26], [31]. Ten studies found that in 8.86% (95% CI 5.68–12.4) of children with probable or confirmed meningitis, *S. pneumoniae* was the causative organism (Figure 2) [10], [12], [14], [15], [17], [21], [23], [25], [26], [28]. Of the positive bacterial isolates from cases of invasive bacterial diseases, 23.74% (95% CI 17.47–30.01) were identified as *S. pneumoniae* by culture, LAT or PCR. Isolates from patients with severe pneumonia and pyogenic meningitis showed that 15.07% (95% CI 8.69–21.45) and 34.23% (95% CI 22.45–46.01) were due to *S. pneumonia*, respectively (Figure 3). Pneumococcus contributed insignificantly to all causes of pneumonia, but was still one of the leading causes of bacterial pneumonia as demonstrated by the meta-analysis.

#### South Asian Countries (Children <5 years of age)

**Figure 4:**
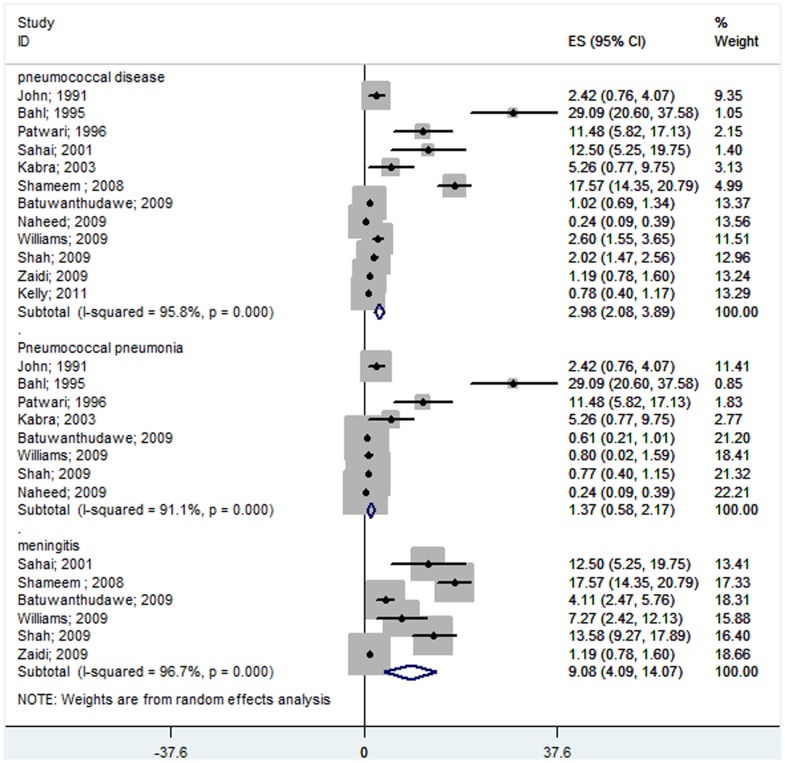
Forest plot showing the proportion of IPD from hospital-based prospective studies in South Asian children under the age of 5 with suspected invasive bacterial disease. The plot also shows a subgroup analysis for the pneumococcal pneumonia cases among all of the pneumonia patients and for the pneumococcal meningitis cases among all of the meningitis patients.

**Figure 5:**
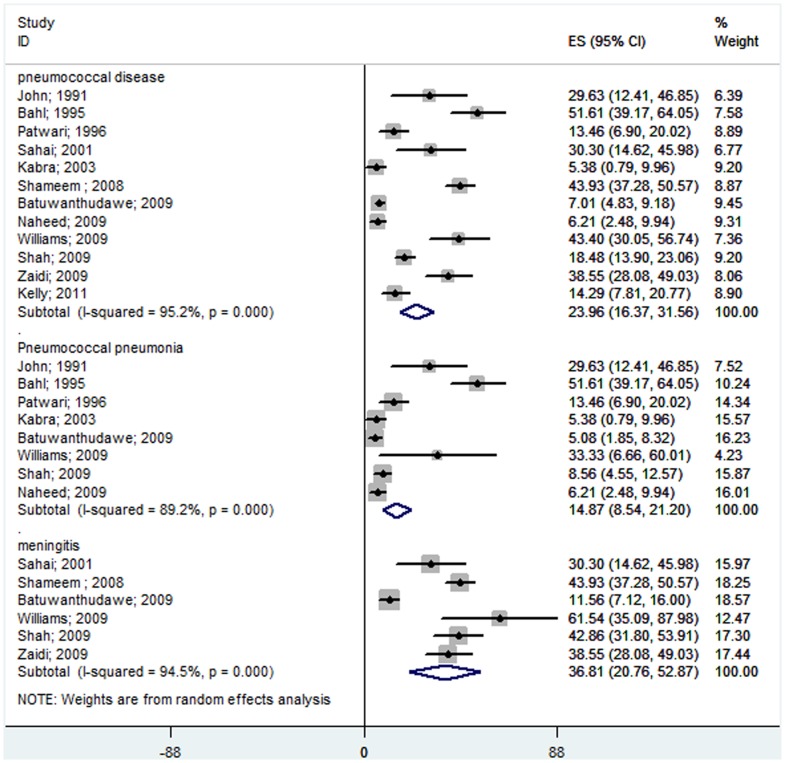
Forest plot showing the proportion of IPD from hospital-based prospective studies in South Asian children less than 5 years of age with confirmed invasive bacterial disease. The plot also shows a subgroup analysis for the pneumococcal pneumonia cases among all the bacterial pneumonia patients and for the pneumococcal meningitis cases among all of the pyogenic meningitis patients.

Of the 16 studies included, only 12 studies [11], [13], [15], [16], [21], [23]–[26], [28], [30], [31] published data on children less than 5 years of age. The meta-analysis showed that 2.98% (95% CI 2.08–3.89%) of children less than 5 years of age who were hospitalized with suspected invasive bacterial disease had *S. pneumoniae* infections. In South Asian children less than 5 years of age, the rate of pneumococcal pneumoniae was 1.37% (95% CI 0.58–2.17%) and the rate of pneumonia and pneumococcal meningitis of all causes was 9.08% (95% CI 4.09–14.07%) (Figure 4). In this age group, pneumococcus caused approximately 24% of all confirmed, invasive, bacterial diseases (Figure 5). Pneumococcal pneumonia was responsible for 14.87% (95% CI 8.54–21.20%) of all cases of bacterial pneumonia, and pneumococcal meningitis was responsible for 36.81% (95% CI 20.76–52.87%) of all cases of pyogenic meningitis (Figure 5).

#### Hospital-based Studies (India)

**Figure 6:**
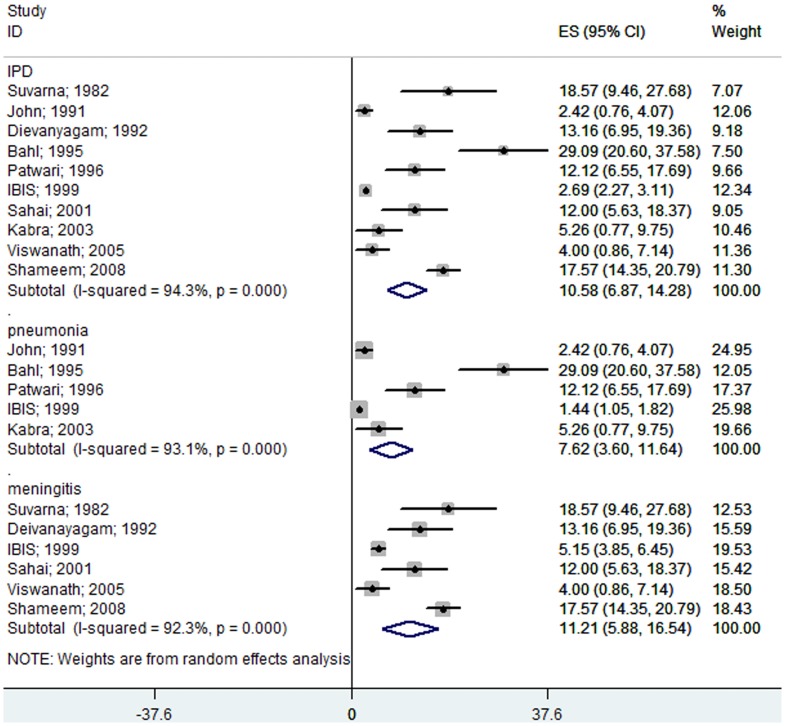
Forest plot showing the proportion of IPD from hospital-based prospective studies in Indian children aged 1 month to 12 years with suspected invasive bacterial disease. The plot also shows a subgroup analysis for the pneumococcal pneumonia cases among all of the pneumonia patients and for the pneumococcal meningitis cases among all of the meningitis patients.

**Figure 7:**
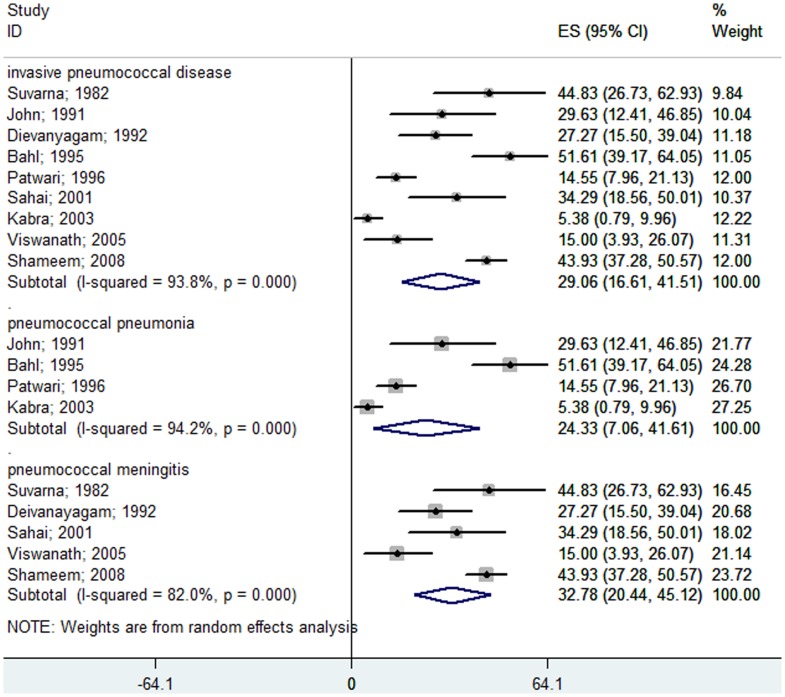
Forest plot showing the proportion of IPD from hospital-based prospective studies in Indian children aged 1 month to 12 years with confirmed invasive bacterial disease. The plot also shows a subgroup analysis for the pneumococcal pneumonia cases among all of the bacterial pneumonia patients and for the pneumococcal meningitis cases among all of the pyogenic meningitis patients

Ten Indian studies [10]–[17], [21], [31] showed that *S. pneumoniae* causes 10.58% (95% CI 6.87–14.28) of invasive diseases, 7.62% (95% CI 3.6–11.64) of severe pneumonia cases, and 11.21% (95% CI 5.88–16.54) of meningitis cases in children with suspected bacterial diseases aged 1 month to 12 years (Figure 6). Streptococcus was a major bacterium isolated from severe pneumonia and pyogenic meningitis cases. *S. pneumoniae* was confirmed in 24.3% (95% CI 7.06–41.61) of bacterial isolates from pneumonia cases and in 32.78% (95% CI 20.44–45.12) of pyogenic meningitis cases (Figure 7).

### Population-based Studies

**Figure 8:**
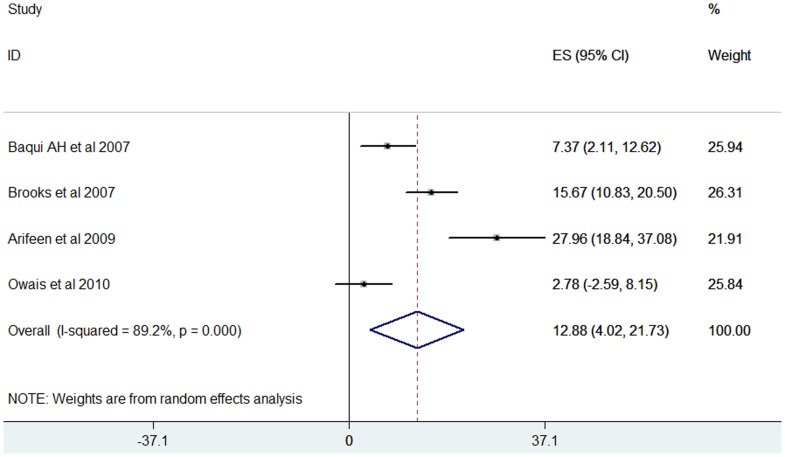
Forest plot showing the proportion of IPD from population-based prospective studies in South Asian children aged 1 month to 12 years with suspected invasive bacterial disease.

Four population-based, active surveillance studies from South Asian countries were included in the review [18], [19], [22], [29]. These studies were conducted in Pakistan and Bangladesh and only examined children less than 5 years of age. These studies showed that *S. pneumoniae* was present in 12.88% (95% CI 4.02–21.73) of all positive bacterial isolates (Figure 8).

### Hospital-based Retrospective Studies

Two hospital-based retrospective studies from India were included in this review [20], [27]. The pooled data showed that *S. pneumoniae* was present in 28.36% (95% CI 22.74–79.46) of isolates from the total number of admitted patients with invasive bacterial diseases.

### Meta-regression Analysis

Table 3 shows the results of the final model in which age (<5 years *versus* any age up to 12 years), setting (hospital *versus* community), diagnostic method (culture *versus* culture + LAT), study centre (multicentre *versus* single site) and organ system (clinical sepsis *versus* specific organ system) were estimated as random effects parameters. Associations between prevalence rates and predictors were assessed using the multivariate method including the predictors that were found to be significant in the univariate meta-regression. The odds of IPD were increased by 9% in studies using culture as well as LAT as diagnostic modalities when compared to studies using culture testing alone, and this association showed a trend toward significance (OR 1.09; 95% CI: 0.99–1.21). Similarly, the IPD prevalence was significantly lowered by 10% in studies where all suspected IPD cases involving any body organ were enrolled in comparison to studies where only subjects with pneumonia or meningitis (i.e., single organ involvement) were investigated (OR 0.90; 95% CI: 0.81–0.99) (Table 3)

**Table 3:** Univariate and multivariate meta-regression of IPD prevalence.

	Odds ratio#	95% CI#	P value#	Odds ratio*	95% CI*	P value*
**Age** (<5 years vs. any age upto 12 years)	0.93	0.82–1.06	0.271			
**Surveillance setting** (Hospital *versus* community),	1.10	0.96–1.26	0.139			
**Diagnostic testing method**(Culture *versus* culture + LAT)	1.11	0.99–1.24	0.066	1.09	0.99–1.21	0.083
**Study centre**(Multicentric *versus* single site)	0.97	0.86–1.10	0.630			
**Organ system**(Any clinical sepsis *versus* specific organ system alone)	0.89	0.80–0.99	0.036	0.9	0.81–0.99	0.046

# for univariate meta-regression; *for multivariate meta-regression.

### Publication Bias

**Figure 9:**
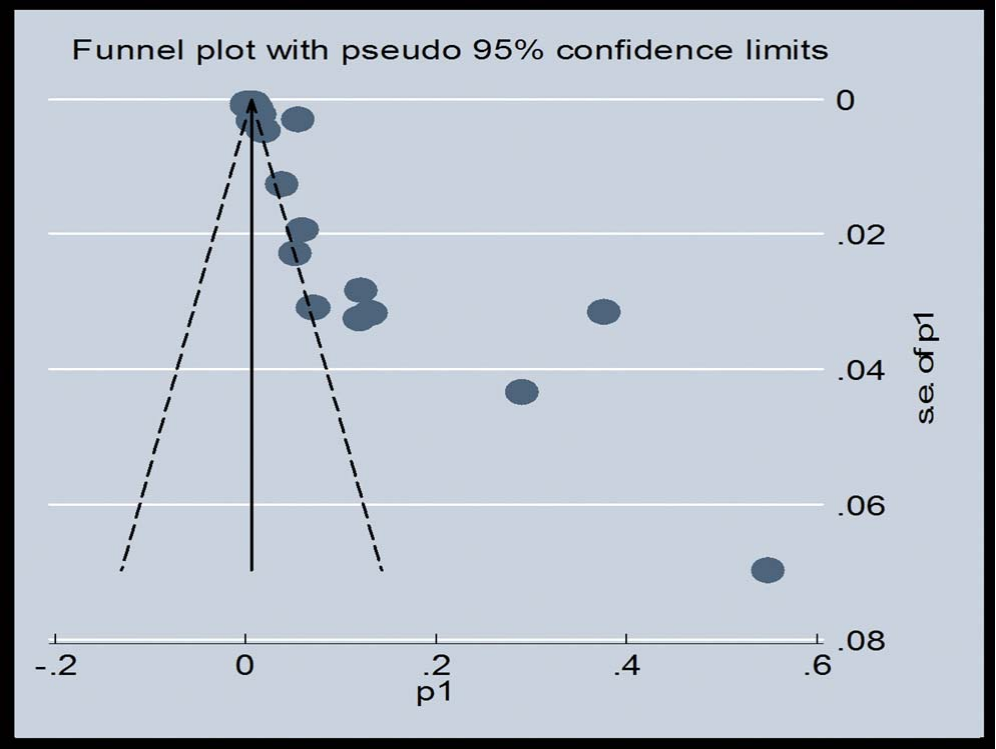
Funnel plot showing the publication bias.

**Figure 10:**
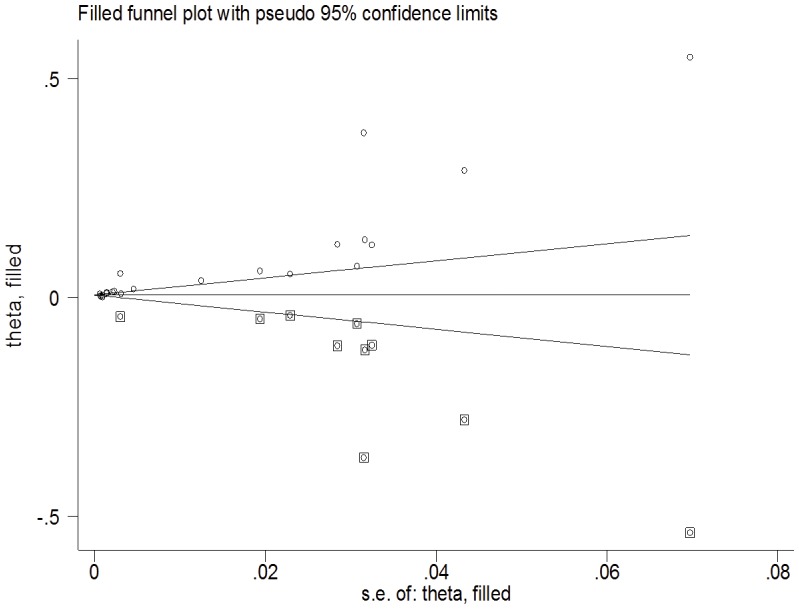
Corrected funnel plot using the trim and fill method.

We used Begg's test to identify any publication bias in the review. A significant *P*-value of 0.003 in the above output of Kendall's tau by Begg's test indicates the presence of publication bias in this meta-analysis. However, Egger's linear regression test showed a nonsignificant coefficient (2.63) of publication bias (95% CI: −3.62 to 8.88) (*P*-value 0.39). We also used the funnel plot (Figure 9) to identify publication bias. The funnel plot was asymmetric and indicated that there were missing studies. Using the trim and fill method in our meta-analysis, it was shown that 10 studies were missing. Under the random-effects model, the point estimate and 95% CI for the pooled prevalence of the combined studies was 2% (1.5–2.5%), which was reduced to 0.09% (0.03–1.5%) using the trim and fill method (Figure 10).

### Heterogeneity

For each trial, the z statistic (effect size/standard error [SE] of effect size) was plotted against the reciprocal SE (1/SE) of the effect size. The unweighted regression line was constrained through the origin with its 95% CI, and it showed a slope equal to the overall pooled prevalence estimate in the fixed effects meta-analysis.

**Figure 11:**
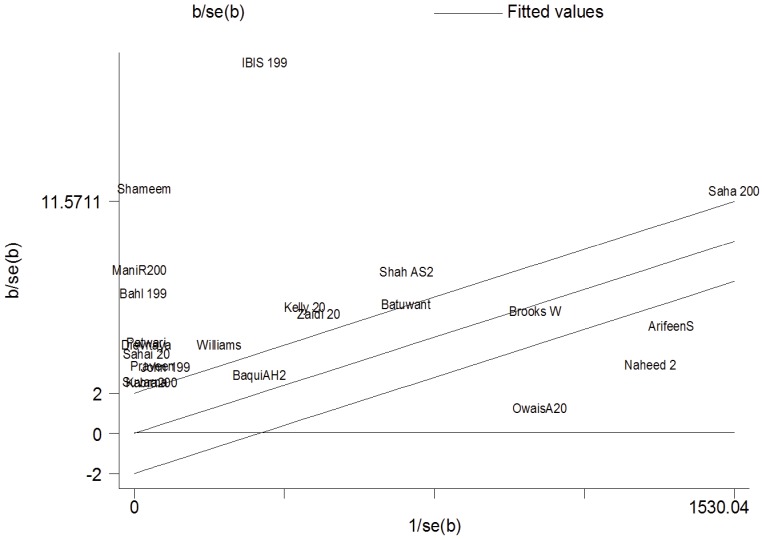
Galbraith plot showing heterogeneity.

The position of each trial on the horizontal axis gives an indication of the weight allocated to it in the meta-analysis. The position on the vertical axis represents the contribution of each trial to the Q statistic for heterogeneity (Figure 11). The results of the I^2^ analysis and Tau^2^ analysis are shown in Table 4.

**Table 4:** Heterogeneity analysis for hospital-based and population-based studies.

	Heterogeneity statistics	Degree of freedom	P	I-squared**	Tau-squared	z test	p value
**Hospital based**	617.12	16	0.000	97.4%	0.0002	8.57	0.000
**Population based**	39.16	4	0.000	89.8%	0.0000	3.51	0.000
**Overall**	684.91	21	0.000	96.9%	0.0001	7.92	0.000

**I-squared: the variation in Effect Size attributable to heterogeneity.

Note: Between-group heterogeneity was not calculated. Significance test(s) of ES  = 0.

## Discussion

The meta-analysis showed that only 1.4% of all severe pneumonia cases in South Asia were due to pneumococcus, which contrasts with the results of other reviews. However, when we examined the bacterial isolates, pneumococcus had a significant contribution in severe pneumonia patients, and it is clearly a major bacterium responsible for severe pneumonia and meningitis. Although several other pathogens including viruses were detected, pneumococcus is an important pathogen that causes severe pneumonia and requires hospitalization.

In India, pneumococcus is a frequent cause of invasive bacterial diseases. It is a common bacterium isolated from children with severe bacterial pneumonia and children with pyogenic meningitis. This meta-analysis demonstrates that IPDs are more common in India than in other South Asian countries.

Although the results come from surveillance studies, which are not generally considered good quality evidence, the studies included in this review were characterized by quality, and they were based on a pretested questionnaire and were rated accordingly. Most of the hospital-based studies were from tertiary care centres where sick and referred patients are admitted, and overestimates are expected. Prior antibiotic use is also common in these patients, which alters the results. In this review, 12 studies reported prior antibiotic usage in 18% of children. This high rate of antibiotic use must have affected the isolation of the causative bacteria. Only the population-based studies from Bangladesh and Pakistan matched the inclusion criteria. Thus, the lack of population-based studies from other countries weakens this meta-analysis. The heterogeneity, due to the different sample sizes, age groups and syndromes investigated in the individual studies, was significant as estimated by the I^2^ and Tau^2^ tests, and thus, a random effects model was used [52].

This is the first systematic review of IPDs in South Asia. The meta-analysis showed that *S. pneumoniae* was the most common bacterium isolated. Though there was a difference in the figures obtained from hospital- and community-based studies, both types of studies reported a high number of cases with *S. pneumoniae* infections.

O' Brien et al. performed a global estimation of the IPD burden and found that India had the highest mortality due to IPD [8]. Rudan et al. showed that India had the highest number of new cases diagnosed worldwide [4]. Johnson et al. tried to determine the most prevalent pneumococcal serotypes globally and found that serotype 14 was the most common cause of IPD in every region, and serotype 1 was common in Asia [53]. Our findings suggest that pneumococcus is the bacterium most commonly isolated by culture, LAT or PCR in fluids from otherwise sterile sites, which suggests that it is the most common aetiologic agent responsible for invasive bacterial diseases in the region. The previous research confirms the same findings using different methods. We analysed the observed frequency of *S. pneumoniae* through a meta-regression framework. In this respect, we accounted for the influence of various covariates on the observed prevalence of *S. pneumoniae*, such as age, diagnostic test used, surveillance setting, study centre and organ system involved.

The results show that pneumococcus poses a significant burden on the healthcare system and also affects the economic condition of patient's family. Steps should be taken to counter this organism, which could involve preventive measures by immunizing against *S. pneumonia* with vaccines like PCV-7, PCV-13 and PCV-10 and also by providing a clean and hygienic environment. Additionally, these steps could involve providing easily approachable healthcare facilities, low-cost antibiotics and home-based follow-ups. Preventive steps should be the first approach. Pakistan is the only country from South Asia to realize the importance of prevention by introducing PCV-10 into its immunization schedule with the help of the GAVI alliance. Bangladesh will also introduce the vaccine shortly. Because India forms the largest part of South Asia and as the data suggests it also forms the largest pneumococcal hub, preventive steps should be initiated in India soon. Most of the Indian data is based on nasopharyngeal sampling, which does not describe the aetiologic role of *S. pneumoniae* but only provides information about its carriage. As described by Johnson et al. [53], PCV-10 or PCV-13 could prevent 80% of all pneumococcal diseases, and a relevant vaccine should be introduced after determining the prevalent serotypes.

Furthermore, increasing drug resistance is creating another problem. Studies from South Asia have shown that there is increasing drug resistance to available antibiotics. In the IndiaCLEN Short Course Amoxycillin Therapy for Pneumonia (**ISCAP**) trial, the resistance pattern of *S. pneumoniae* to various antibiotics was: cotrimoxazole 66.3%, chloramphenicol 9.0%, oxacillin 15.9% and erythromycin 2.8% [54]. The Asian Network for Surveillance of Resistant Pathogens (ANSORP) study reported that 41% of strains were not susceptible to penicillin in Sri Lanka, and this rate was approximately 4% in India [55]. Kunango et al. reported that out of 150 clinical isolates from invasive pneumococcal infections, only 11 (7.3%) isolates were relatively resistant to penicillin, although 64 were resistant to one or more antibiotics, especially cotrimoxazole, tetracycline and chloramphenicol [56]. Unpublished data from one site of a multicentric trial IndiaCLEN severe pneumonia oral therapy (ISPOT) study in India showed that in approximately 38% of children with severe radiologically-confirmed pneumonia, *S. pneumoniae* was isolated from the nasopharyngeal aspirates or throat swabs. The study also showed that oral amoxicillin administered at home was effective in treating severe pneumonia. The No Shots study from Pakistan concluded that home treatment with high-dose oral amoxicillin in cases of severe pneumonia is equivalent to WHO recommendations [57]. Similarly, another study from Pakistan showed that local health workers were able to treat severe pneumonia cases at home with high-dose amoxicillin [58]. Moreover, Das and Singh showed that oral amoxicillin administered either in a hospital or in a community setting was effective in treating severe pneumonia and was not inferior to the standard treatment [59].

The authors strongly feel that there is a need for the introduction of a pneumococcal conjugate vaccine in South Asian countries. The prevalent serotypes should first be determined in order to introduce a relevant vaccine. Drug resistance should be assessed, and treatment guidelines should then be revisited in view of increasing drug resistance.

## Supporting Information

Checklist S1 **PRISMA checklist.**
(DOCX)

Appendix S1 **Detailed Search strategy.**
(DOCX)
